# Efficient Degradation of High-Molecular-Weight Hyaluronic Acid by a Combination of Ultrasound, Hydrogen Peroxide, and Copper Ion

**DOI:** 10.3390/molecules24030617

**Published:** 2019-02-11

**Authors:** Hongyue Chen, Jing Qin, Yi Hu

**Affiliations:** School of Pharmaceutical Sciences, State Key Laboratory of Materials-Oriented Chemical Engineering, Nanjing Tech University, Nanjing 210009, China; hongyuechen@njtech.edu.cn (H.C.); 904536252@njtech.edu.cn (J.Q.)

**Keywords:** hyaluronic acid, degradation, ultrasound, spectroscopic characterization

## Abstract

Hyaluronic acid (HA) was depolymerized by a combination of ultrasound, hydrogen peroxide and copper ion. The structures of high-molecular-weight hyaluronic acid (HMW-HA) and low-molecular-weight hyaluronic acid (LMW-HA) were determined by Fourier transform infrared (FTIR) spectroscopy, circular dichroism (CD) spectroscopy, and UV-VIS absorption spectroscopy. The degradations of HMW-HA using a physical method, a chemical method, and a combination of physical and chemical method were compared. The results show that HA can be effectively degraded by a combinatorial method involving ultrasound, hydrogen peroxide, and copper ion. Under the degradation conditions of 50 mM H_2_O_2_, 5.0 μM CuCl_2_, 160 W, pH 4.0, and reaction at 50 °C for 30 min, the content of glucuronic acid was 36.56%, and the yield of LMW-HA was 81.71%. The FTIR, CD, and UV-VIS absorption spectra of HA did not change with the decrease in molecular weight, indicating that the structure of HA remained intact during the degradation.

## 1. Introduction

Hyaluronic acid (HA) is a linear polysaccharide consisting of two alternating units, β-1,4-d-glucuronic acid and β-1,3-*N*-acetyl-d-glucosamine; HA is the main component of extracellular matrix [1,2]. HA is also present in the skin, joints, and cornea at high concentrations. HA is a homogeneous polymer, and the molecular size is widely distributed (10^5^–10^7^ Da). High-molecular-weight hyaluronic acid (HMW-HA) inhibits cell proliferation, migration of vascular endothelial cell, and angiogenesis, whereas low-molecular-weight hyaluronic acid (LMW-HA) with completely different physiological functions promotes the adhesion and proliferation of endothelial cells [3,4,5,6].

The molecular weight of hyaluronic acid usually exceeds 10^6^ Da. Various methods have been reported to produce LMW-HA, including chemical degradation, physical degradation, and enzymatic degradation [7,8,9]. Methods of chemical degradation mainly include acid hydrolysis, alkaline hydrolysis, and oxidative degradation [10,11,12]. Acid, alkali, and oxidant react with HMW-HA at different reaction sites. Thus, the glycosidic bond is broken, and HA is degraded. In some oxidative degradation systems, metal catalysts are also added to increase the activity of radicals such as OH·, O·, and H·. Using a system containing H_2_O_2_ plus CuCl_2_, the dynamic viscosity of the HA solution indicated that the degradation of biopolymer decreased faster than the system containing only H_2_O_2_ [13]. Ultrasonication is a common method of physical degradation to obtain LMW-HA. Ultrasonic irradiation may decrease the molecular weight, viscosity, molecular radius, and pH of HA [14]. In this study, H_2_O_2_, CuCl_2_, and ultrasonication were first combined and used to degrade HMW-HA. The molecular weight of HA was measured by size exclusion chromatography coupled with a multiangle laser light scattering photometer (SEC-MALLS). The effects of degradation methods on the HA structure were observed by FT-IR, circular dichroism (CD), and UV-VIS absorption spectroscopy.

## 2. Results and Discussion

### 2.1. Effects of Different Degradation Conditions

#### 2.1.1. Effects of Hydrogen Peroxide Concentration on HA Degradation

In the reaction system, hydrogen peroxide would produce free radicals such as H·, O·, and OH·. Through the action of free radicals with the molecular chains of HA, the reaction sites are activated, leading to the cleavage of glycosidic bond to degrade HMW-HA [15].

Six different concentrations of hydrogen peroxide were screened to catalyze this degradation reaction, and the results are shown in Table 1. A total of 50 mM (Table 1, entry 5) was identified to be the best concentration of hydrogen peroxide to promote the degradation of HMW-HA by comparing the weight-average molecular weight of LMW-HA. The values of dispersity indices (Mw/Mn) range from 1.063 to 1.106, i.e., the molecular weight distribution of LMW-HA is narrow.

#### 2.1.2. Effects of Copper Ion Concentration on HA Degradation

Hydrogen peroxide plus copper ions is an efficient system for the degradation of high-molecular-weight polysaccharides. In the presence of copper ions, the efficiency of hydrogen peroxide producing free radicals is greatly improved. The reaction mechanism suggests that hydrogen peroxide and copper ions would form peroxo–metal complexes; subsequent self-decomposition results in oxygen evolution [16]. The reactions of hydrogen peroxide and copper ions can be expressed as follows:

(1) $$2Cu^{2+}+H_{2}O_{2}\to 2Cu^{+}+O_{2}+2H^{+}$$

(2) $$Cu^{+}+H_{2}O_{2}\to Cu^{2+}+\cdot OH+OH^{-}$$

(3) $$Cu^{+}+O_{2}\to Cu^{2+}+{O_{2}}^{\cdot -}$$

(4) $${O_{2}}^{\cdot -}+H^{+}↔\cdot OOH$$

(5) $$Cu^{+}+2Cl^{-}\to {\left[CuCl_{2}\right]}^{-}$$

(6) $$2H_{2}O_{2}\to \cdot OH+\cdot OOH+H_{2}O$$

H_2_O_2_ acts as a reductant, and Cu^2+^ is reduced to Cu^+^ with the production of oxygen (Equation (1)). Then, Cu^+^ immediately reacts with H_2_O_2_ in a Fenton-like reaction (Equation (2)). It can also be re-oxidized by oxygen to produce superoxide anion (Equation (3)) and subsequent hydroperoxyl radicals (Equation (4)). Cu^+^ is stable in the reaction system because of the presence of chloride ions (Equation (5)). The combination of Equations (1)–(4) describes the formation of reactive radical species (Equation (6)).

Different concentrations of copper ions were studied to increase the degradation rate of HMW-HA. As shown in Table 2, with the increase in the concentration of CuCl_2_, the HA degradation effect was better within certain limits. This clearly shows that 5.0 μM CuCl_2_ had the best catalytic efficiency for the degradation of HMW-HA.

#### 2.1.3. Effects of pH on HA Degradation

Five different pH values were screened; the results are shown in Table 3. As shown in Table 3, when pH is equal to 4, the degradation rate of HMW-HA is the fastest. Above pH 4.0, the higher the pH, the lower the degradation efficiency of HMW-HA. Studies have shown that pH affects the stability of hydrogen peroxide. When the pH increases, the stability of hydrogen peroxide decreases. Under alkaline conditions, hydrogen peroxide may decompose rapidly, thus decreasing its ability to produce free radicals; therefore, the degradation efficiency of hyaluronic acid was reduced.

#### 2.1.4. Effects of Temperature on HA Degradation

To rule out the effect of temperature on the experiment, temperatures of 20, 30, 40, 50, and 60 °C were investigated. The results are shown in Table 4. The degradation rate increased as the temperature increased. The molecular weight of LMW-HA obtained at a reaction temperature of 50 °C was not different from that obtained at a reaction temperature of 60 °C. A very high temperature will affect the stability of hydrogen peroxide. Finally, 50 °C was selected for the degradation experiments.

#### 2.1.5. Effects of Ultrasonic Power on HA Degradation

Ultrasound is a conventional method to obtain low-molecular-weight polymers by degrading high-molecular-weight polymers. Many mechanisms have been proposed; recent studies show that the main mechanism of ultrasonic depolymerization involves mechanical bond breaking and free radical redox reactions [14,17]. The propagation of sound energy causes a rapid pressure change, thus creating small bubbles in the liquid. The formation and subsequent collapse of bubbles produce intense and rapid change in mechanical motion, and then the molecules in the medium are degraded by the action of high-speed vibration and shear force. Mechanical effects that increase with the increase in relative molecular mass of substances are more significant for polymer substances. A free-radical REDOX reaction is mainly caused by the cavitation effect of liquid under ultrasonic wave. Free radicals and thermal effects are more effective for relatively low-molecular-weight substances. Four different ultrasonic powers were examined. The results shown in Table 5 clearly indicate that 160 W was the best ultrasonic power to promote the degradation of HMW-HA. Based on the above investigations, the optimal degradation conditions were 50 mM H_2_O_2_, 5.0 μM CuCl_2_, pH 4.0, 160 W ultrasonic power, and the reaction temperature 50 °C.

#### 2.1.6. Comparison of Degradation Efficiency of Hyaluronic Acid under Different Conditions

**Figure 1:**
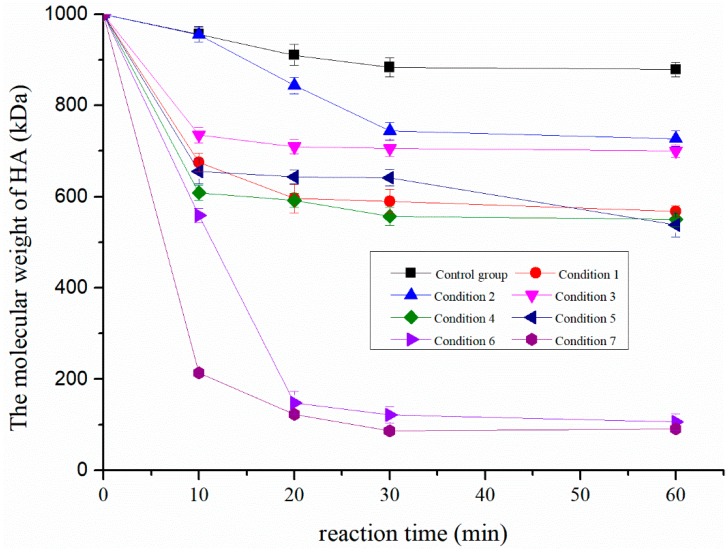
Comparison of degradation efficiency of hyaluronic acid under different conditions.

As shown in Figure 1, seven different degradation conditions were compared. Ultrasound (condition 1), copper ions (condition 2), and hydrogen peroxide (condition 3). All of them had catalytic effects on the degradation of hyaluronic acid. The combination of ultrasound and copper ions (condition 4), ultrasound and hydrogen peroxide (condition 5), and hydrogen peroxide and copper ions (condition 6) had synergistic effects on the degradation of HA. Among the seven conditions, the catalytic efficiency of condition 6 was the best. However, the combination of ultrasound, hydrogen peroxide, and copper ion (condition 7) showed better degradation efficiency; 30 min later, LMW-HA with a molecular weight of 74.9 kDa was obtained. This is probably because ultrasound promotes the production of free radicals to some extent [18], and when the molecular weight of hyaluronic acid is relatively high, the mechanical effect of ultrasound on HMW-HA is significant.

### 2.2. Structural Analysis of LMW-HA

#### 2.2.1. Content of Glucuronic Acid in LMW-HA

**Figure 2:**
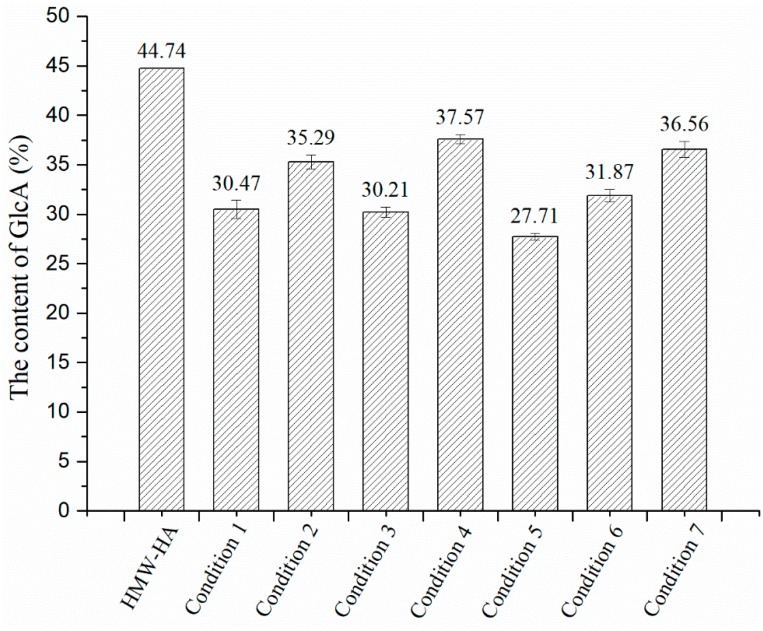
Content of glucuronic acid in LMW-HA degraded under each of seven conditions.

Bitter–Muir method was used to measure the content of glucuronic acid in LMW-HA, which was degraded under the seven conditions described in Section 3.3.6 for 30 min. The results are shown in Figure 2. The content of glucuronic acid in LMW-HA degraded under the optimal degradation condition was 36.56%, slightly lower than the content of glucuronic acid in LMW-HA, which was 44.74%. This indicates that the sugar ring of HA is slightly damaged during the degradation. The yield of degraded HA was 81.71%, slightly higher than the content of glucuronic acid (77.43%) in LMW-HA degraded using a combinatorial method of microwave, hydrogen peroxide, and ascorbic acid [19]. Figure 2 shows that although condition 6 also had a high degradation efficiency, the content of glucuronic acid in the degradation products was lower than that in condition 7, indicating that the combination of ultrasound, hydrogen peroxide, and copper ions in this research could significantly improve the degradation efficiency of HMW-HA and helped to maintain the integrity of product structure.

#### 2.2.2. FTIR Spectra of HA and LMW-HA

**Figure 3:**
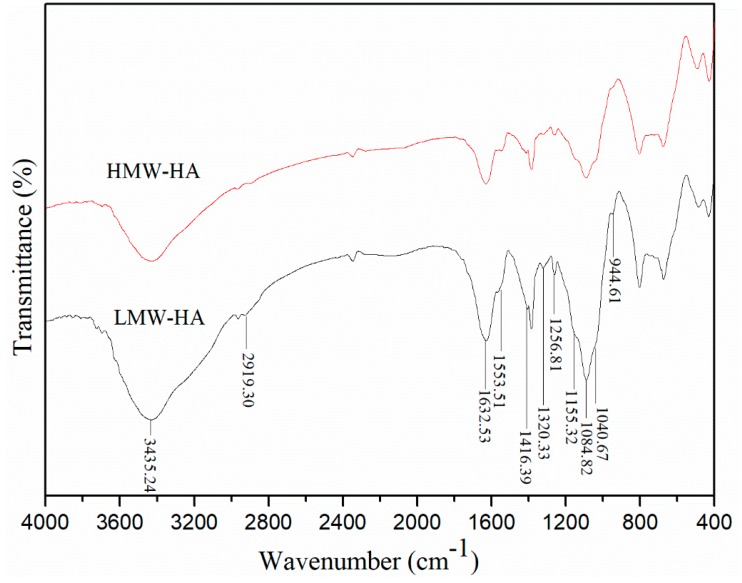
FTIR spectra of intact HA and LMW-HA degraded using the optimal method.

Figure 3 shows FTIR spectra of HMW-HA and LMW-HA obtained using the final degradation method. No obvious difference was observed from the FTIR spectra of HMW-HA and LMW-HA, i.e., the structure of LMW-HA was not affected by the degradation method. A strong absorption band was observed at 3435.24 cm^−1^, indicating OH and NH stretching vibrations; the band at 2919.30 cm^−1^ indicates CH symmetrical and CH_2_ asymmetrical stretching; the band at 1256.81 cm^−1^ can be attributed to *v*(C-O) (of COOH). The absorption bands at ~1155.32 cm^−1^, 1084.82 cm^−1^, 1040.67 cm^−1^, and 944.61 cm^−1^ are typical for carbohydrates, and the bands at 1632.53 cm^−1^, 1553.51 cm^−1^, and 1320.33 cm^−1^ can be assigned to amide I, II, and III. The band at 1416.39 cm^−1^ is assigned to symmetric C-O stretching vibrations [20].

#### 2.2.3. CD Spectrum of HA and LMW-HA

**Figure 4:**
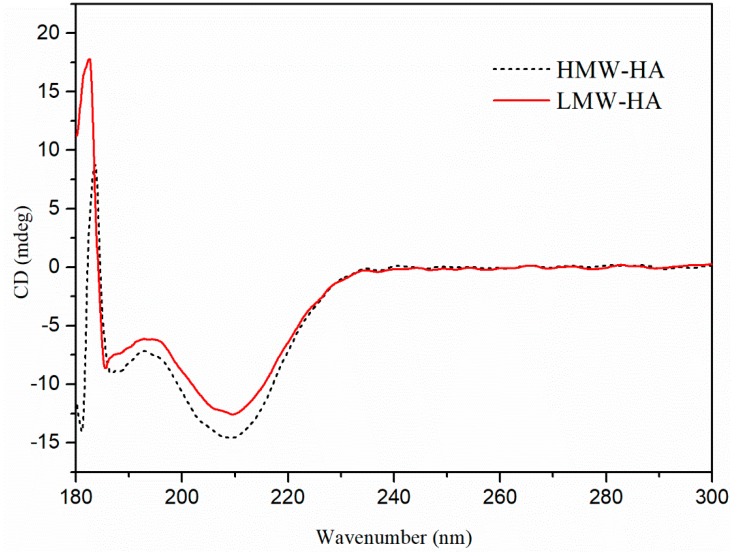
CD spectra of HMW-HA and LMW-HA.

The CD spectra of HMW-HA and LMW-HA obtained under the optimal degradation conditions are shown in Figure 4; no obvious difference was observed. The CD spectrum of polysaccharides is related to the molecular structure and conformation. The chiral double helix structure of a CD spectrum is very sensitive, and the negative peak in a CD spectrum indicates that the molecule has β-sheet conformation. The 183 nm band could be attributed to the carboxyl π–π* transition in the molecule, the negative peak at 187 nm could be attributed to the π–π* transition of GlcNAc in HA, and the negative peak at 210 nm corresponds to the n–π* transition of carboxyl group [21]. The results of CD spectra are consistent with the results of FTIR spectra and the determination of GlcA content, indicating that the structure of LMW-HA remained intact and unchanged during the degradation.

#### 2.2.4. UV–VIS Absorption Spectra

**Figure 5:**
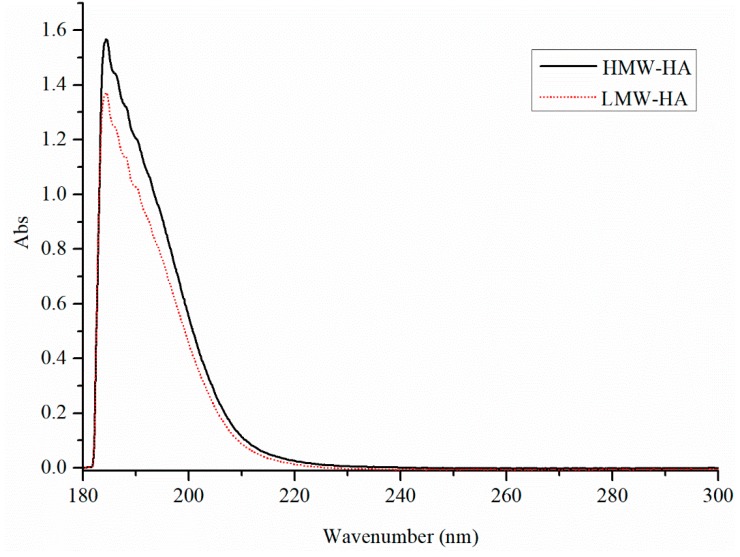
UV–VIS absorption spectra of HMW-HA and LMW-HA.

The high similarity between the UV spectra of HMW-HA and LMW-HA (see Figure 5) suggests that the structure of HA was not damaged during the degradation under the optimal degradation conditions, similar to the result reported by Wu [22].

## 3. Materials and Methods

### 3.1. Materials

The high-molecular-weight HA sample (1000 kDa) used throughout the study was purchased from Bloomage Freda Biopharm Co. Ltd. (Shandong, China) NaCl and NaOH were purchased from Xilong scientific (Guangzhou, China). CuCl_2_·2H_2_O was purchased from Energy Chemical (Shanghai, China); hydrochloric acid and H_2_O_2_ were purchased from Shanghai Lingfeng Chemical Reagent Co. Ltd (Shanghai, China). All the chemicals were of analytical grade.

### 3.2. Analytical Methods

The molecular weight of HA fragment was determined by SEC-MALLS. The chromatography system consisted of an AKTA Purifier pump (P-900), a rheodyne injection valve (INV-907) fitted with a 100 μL loop and Shodex SB-804-HQ column. Eluent identical to the solvent used to dissolve the samples was monitored using a DAWN EOS Multiangle Laser Light Scattering Instrument (Wyatt Technology, Santa Barbara, CA, USA) and RefractoMax521 Refractive Index Detector (Thermo Scientific, Waltham, MA, USA). The refractive index increment (dn/dc) was 0.150 mL/g [23]. Vacuum degassed mobile phase aqueous 0.9% NaCl solution was pumped at a flow rate of 0.5 mL/min. Samples were dissolved in the mobile phase aqueous solution to the required concentration. Prior to analysis, the samples were filtered through a 0.22 μm nylon filter. Signals from the Multiangle Laser Light Scattering Instrument and Refractive Index Detector were captured and analyzed using ASTRA software (Version 5.3.4, Wyatt Technology, Santa Barbara, CA, USA) supplied by the manufacturer.

Bitter–Muir method to measure the content of glucuronic acid could be used to determine the hyaluronic acid content as 100 g hyaluronic acid contained about 46.32 g glucuronic acid [24]. FT-IR spectra were obtained in the transmission mode using a Thermo NICOLET IS 10 infrared spectrophotometer in the range of 400–4000 cm^−1^. Samples were prepared as a film using the KBr pellet technique. CD spectra were measured using a JASCOJ810 spectropolarimeter (Jasco, Tokyo, Japan). Samples were dissolved in deionized water at a concentration of 0.5 mg/mL. The optical path of quartz sample cell was 0.1 cm. The range of scanning wavelength was 180–300 nm, and the scanning speed was 50 nm min^−1^. Baseline correction was automatically carried out with the solvent (deionized water) as blank group. UV–VIS absorption spectra were measured using a JASCOJ810 spectropolarimeter (Jasco, Tokyo, Japan) in the range from 180 nm to 300 nm. Samples were diluted in distilled water at 0.5 mg/mL, and distilled water was used as the reference.

### 3.3. Degradation Studies

In the preliminary work, the components of oxidative system, pH, temperature, and the power of ultrasound showed significant influence on the degradation of HMW-HA. A series of experiments were carried out to determine the best degradation conditions.

#### 3.3.1. Effect of Hydrogen Peroxide Concentration

A total of 0.5% (*w*/*v*) HMW-HA solution was prepared by dissolving in 0.2 M NaCl solution and gently stirring at room temperature overnight in the dark. Six different oxidative systems were prepared with 0.1 μM CuCl_2_ and different concentrations of hydrogen peroxide: (1) 10 mM H_2_O_2_, (2) 20 mM H_2_O_2_, (3) 30 mM H_2_O_2_, (4) 40 mM H_2_O_2_, (5) 50 mM H_2_O_2_, and (6) 60 mM H_2_O_2_. The reaction temperature was adjusted to 40 °C; pH was 6.5; the ultrasonic power was 160 W; the reaction time was 30 min. The reaction products were precipitated in twice the volume of ethanol; then, the polymer precipitate was washed with 20 mL ethanol, centrifuged, and dried in a vacuum drying oven for the following studies.

#### 3.3.2. Effect of CuCl_2_ Content

The reaction conditions were the same as 3.3.1, except for the content of hydrogen peroxide and CuCl_2_. The concentration of hydrogen peroxide was 50 mM, and the concentrations of CuCl_2_ were (1) 0.1 μM, (2) 1.0 μM, (3) 2.0 μM, (4) 3.0 μM, (5) 4.0 μM, (6) 5.0 μM, and (7) 6.0 μM.

#### 3.3.3. Effect of pH

In the previous study, the optimal concentrations of hydrogen peroxide and CuCl_2_ were determined. In this section, the effect of pH on HMW-HA degradation was evaluated. The values of pH were 3, 4, 6, 8, and 10.

#### 3.3.4. Effect of Temperature

Based on the previous study, the effect of temperature on HMW-HA degradation was evaluated, and the temperatures were 20 °C, 30 °C, 40 °C, 50 °C, and 60 °C.

#### 3.3.5. Effect of Ultrasonic Power

Through the previous study, the optimal conditions such as pH, temperature, and concentrations of hydrogen peroxide and CuCl_2_ were determined. Consequently, the effect of ultrasonic power on HMW-HA degradation was evaluated, and the values of ultrasonic power were set at 135 W, 150 W, 160 W, and 200 W.

#### 3.3.6. Comparison of Degradation Efficiency of HMW-HA under Different Conditions

To confirm the high efficiency of HMW-HA degradation by a combination of ultrasound, hydrogen peroxide, and copper ion, the degradation effects of different methods were compared under the optimal reaction conditions.

Condition 1: HA solution was degraded using 160 W ultrasound at 50 °C. The solution pH was 4. The reaction times were 10 min, 20 min, 30 min, and 60 min.

Condition 2: HA solution was degraded using 5.0 μM CuCl_2_ at 50 °C. The solution pH was 4. The reaction times were 10 min, 20 min, 30 min, and 60 min.

Condition 3: HA solution was degraded using 50 mM H_2_O_2_ at 50 °C. The solution pH was 4. The reaction times were 10 min, 20 min, 30 min, and 60 min.

Condition 4: HA solution was degraded using 5.0 μM CuCl_2_ with 160 W ultrasound at 50 °C. The solution pH was 4. The reaction times were 10 min, 20 min, 30 min, and 60 min.

Condition 5: HA solution was degraded using 50 mM H_2_O_2_ with 160 W ultrasound at 50 °C. The solution pH was 4. The reaction times were 10 min, 20 min, 30 min, and 60 min.

Condition 6: HA solution was degraded using 50 mM H_2_O_2_ with 5.0 μM CuCl_2_ at 50 °C. The solution pH was 4. The reaction times were 10 min, 20 min, 30 min, and 60 min.

Condition 7: HA solution was degraded by 160 W ultrasound, 50 mM H_2_O_2_, and 5.0 μM CuCl_2_ at 50 °C. The solution pH was 4. The reaction times were 10 min, 20 min, 30 min, and 60 min.

## 4. Conclusions

In this study, physical and chemical methods were combined to degrade HMW-HA. The effect of ultrasonic power, concentrations of hydrogen peroxide and copper ions, pH, and reaction temperature on hyaluronic acid degradation was investigated. Under the optimal degradation conditions of 50 mM H_2_O_2_, 5.0 μM CuCl_2_, pH 4.0, and reaction temperature at 50 °C for 30 min, the yield of the degraded HA was 81.71% with 36.56% glucuronic acid content. The results show that a combination of ultrasound, hydrogen peroxide, and copper ion is a novel and efficient degradation method for the preparation of LMW-HA. The FTIR, CD, and UV–VIS absorption spectral characterizations also confirm that the structure of LMW-HA remained intact during the degradation.

## Figures and Tables

**Table 1:** Average molecular weights and dispersity indices of LMW-HA under different concentrations of hydrogen peroxide.

Entry	Hydrogen Peroxide Concentration (mM)	Mn (kDa)	Mw (kDa)	Mw/Mn
1	10	560.1	619.3	1.106
2	20	501.8	533.2	1.063
3	30	508.7	544.3	1.07
4	40	431.6	464.6	1.076
5	50	414.2	446.5	1.078
6	60	472.2	507.2	1.074

The averages of molar mass—numeric: Mn and weight: Mw, dispersity indices (Mw/Mn).

**Table 2:** Average molecular weights and dispersity indices of LMW-HA under different concentrations of copper ions.

Entry	CuCl_2_ Concentration (μM)	Mn (kDa)	Mw (kDa)	Mw/Mn
1	0.1	413.2	449.3	1.087
2	1.0	374.6	405.3	1.082
3	2.0	311.5	348.6	1.119
4	3.0	206.4	256.9	1.245
5	4.0	86.06	123.5	1.435
6	5.0	87.58	111.5	1.273
7	6.0	83.47	112.8	1.351

The averages of molar mass—numeric: Mn and weight: Mw, dispersity indices (Mw/Mn).

**Table 3:** Average molecular weights and dispersity indices of LMW-HA under different pH.

Entry	pH	Mn (kDa)	Mw (kDa)	Mw/Mn
1	3	82.71	126.3	1.527
2	4	72.46	102.3	1.412
3	6	90.17	118.0	1.309
4	8	248.8	374.6	1.506
5	10	220.0	322.9	1.468

The averages of molar mass—numeric: Mn and weight: Mw, dispersity indices (Mw/Mn).

**Table 4:** Average molecular weights and dispersity indices of LMW-HA at different temperatures.

Entry	Temperature (°C)	Mn (kDa)	Mw (kDa)	Mw/Mn
1	20	65.74	101.2	1.539
2	30	85.56	120.4	1.407
3	40	70.89	110.6	1.560
4	50	47.98	71.70	1.494
5	60	45.35	68.05	1.500

The averages of molar mass—numeric: Mn and weight: Mw, dispersity indices (Mw/Mn).

**Table 5:** Average molecular weights and dispersity indices of LMW-HA under different ultrasonic powers.

Entry	Ultrasonic Power (W)	Mn (kDa)	Mw (kDa)	Mw/Mn
1	135	91.11	122.1	1.340
2	150	80.34	108.7	1.353
3	160	51.67	74.9	1.449
4	200	81.84	114.6	1.400

The averages of molar mass—numeric: Mn and weight: Mw, dispersity indices (Mw/Mn).
